# P-1237. Comparison of Non-trapezoidal and Trapezoidal Pharmacokinetic Equations to Calculate Vancomycin Area Under the Curve within 24 Hours of Therapy in Patients with Persistent Methicillin-Resistant *Staphylococcus aureus* Bacteremia

**DOI:** 10.1093/ofid/ofae631.1419

**Published:** 2025-01-29

**Authors:** Abdulwhab Shremo Msdi, Karen K Tan, Jacinda C Abdul-Mutakabbir

**Affiliations:** Loma Linda Medical Center, Riverside, California; Loma Linda University Medical Center, Loma Linda, California; University of California San Diego, San Diego, CA

## Abstract

**Background:**

Achieving target vancomycin (VAN) area under the curve within 24 hours of therapy (AUC_0-24_) is associated with better clinical outcomes for serious methicillin-resistant *Staphylococcus aureus* (MRSA) infections. Two first-order pharmacokinetic (PK) equations, e.g. the trapezoidal and non-trapezoidal methods, can be used to estimate AUC. The 2020 vancomycin monitoring guidelines do not address which PK equation to estimate AUC_0-24_. Furthermore, there is limited data directly comparing these methods on day 1 of therapy, especially in patients with confirmed MRSA infection. This study aims to compare the calculated VAN AUC_0-24_ using the two PK equations in patients with persistent MRSA bacteremia. Then, compare the first-order PK computed values to a Bayesian-calculated AUC_0-24_.

**Methods:**

This was a retrospective cohort study of adult patients with persistent MRSA bacteremia (MRSAB) for ≥ 3 days between December 1^st^ 2020, to December 31^st^ 2023. Patients treated with intravenous vancomycin for ≥ 48 hours and had two consecutive serum VAN concentrations collected within 48 hours of therapy were screened for study inclusion. AUC_0-24_ was calculated using first-order PK equations (e.g. non-trapezoidal, trapezoidal), and Bayesian software (DoseMeRx). Spearman correlation coefficient (rs) was used to assess correlations between linear PK equations and Bayesian software.

**Results:**

A total of 79 patients with MRSAB were screened, 12 of whom had a persistent MRSAB. The median duration of bacteremia was 7 days (4, 11). The median AUC_0-24_ estimated by non-trapezoidal and trapezoidal methods were 605 mg*h/l (IQR 403, 484) and 453 mg*h/L (IQR 356, 707), respectively. A stronger correlation was observed between AUC_0-24_ estimated by the non-trapezoidal method and Bayesian (rs = 0.82; P 0.001), compared to the trapezoidal method and Bayesian (rs = 0.61; P 0.035).

**Conclusion:**

Both first-order PK equations correlated with Bayesian-estimated AUC_0-24_, with a stronger trend towards non-trapezoidal method. Further research is needed to corroborate this finding.

**Disclosures:**

**Jacinda C. Abdul-Mutakabbir, PharmD, MPH**, CSL Sequiris: Advisor/Consultant|CSL Sequiris: Honoraria|GSK: Advisor/Consultant|GSK: Honoraria|Shionogi: Advisor/Consultant|Shionogi: Honoraria

